# Supplementary material for: Nutritional quality of selected commercially available seed oils and effect of storage conditions on their oxidative stability
Source: PLoS One. 2024 Oct 4;19(10):e0308117. doi: 10.1371/journal.pone.0308117 (PMC11452028; doi:10.1371/journal.pone.0308117)
Supplement: Attachment — Submitted filename: Response to Reviewers.docx [file pone.0308117.s003.docx]

**Rebuttal Letter**

The manuscript has been carefully revised and improved in all aspects including its grammar, clarity and language. All the comments/reservations have been fully addressed. All the changes have been marked with blue font in the manuscript.

**Reviewer #1**

**Comment 1:** The Title may be revised as the nutritional compositions of the oils were only determined in raw materials before storage.

**Author’s response:** We appreciate the reviewer's insightful suggestion and revised the title which is now more reflection of the results. The revised title is “Nutritional quality of selected commercially available seed oils and effect of storage conditions on their oxidative stability”

**Comment 2:** The abstract should be revised. Some important information must be covered and reflect the main findings according to the objectives. Lines 21, the seed oils used in this study should be specified, what are they? Short information about methodology must be included as the abstract should be stand-alone. Line 23 should be mentioned the values of all seed oils. Reorganize the writing, the results in the abstract should be written in the same order as in the results section. Lines 34-35 should be revised please see the details and comments provided in the conclusion section.

**Author’s response:** We appreciate the reviewer's all insightful suggestions regarding improvement of abstract. The abstract is revised as per guidelines and the methodology and results are added in the abstract now as per suggestion.

**Comment 3: Introduction section**

3.1 Lines 76-82 should be deleted. It has already been mentioned in the methodology. 3.2 The literature review is insufficient. A background information and literature review on the effect of improper environmental conditions on oil quality and chemical compositions reported by previous studies are required.

Author’s response: The introduction section is revised, and the more relevant literature is added. The repeated sentences are also deleted as suggested. All the changes have been marked with blue font.

**Comment 4: Materials and methods:** 4.1 Lines 145-151, please revise and detail the storage conditions. The room temperature should be in range, not a single temperature. How to get the oils to explode to light and absent to light? What kind and intensity of light? How to control temperature during storage at high temp?

**Author’s response:** The section is revised as per guidelines. The detail of sample storage is added in the section. The range of room temperature (25-29 ^o^C) is added. Samples were exposed to daylight in a transparent glass container while for the storage in dark, the samples were kept in amber glass bottles and covered with aluminum foil to avoid light. Moreover, the storage temperatures were maintained in glass door hot air ovens.

**Comment 5: Results and discussion:** More discussion on section FA composition, tocopherols, TPC, and TFC are required. The values of these parameters obtained in this study could be both higher and lower than previous studies or even like other studies, so authors need to discuss all sides. More citations are required. From the values of FFA, PV, and PAV, authors should also discuss the standard values of these parameters, as long as these values obtained do not exceed the standard values, these would be accepted. In Figures 1, 2, and 3, the error bars or superscripts should be inserted to indicate statistical differences.

Author’s response: Thank you so much for your valuable suggestions. We have updated discussion of results of the above-mentioned sections mainly fatty acid composition, tocopherols TPC and TFC. Now results have been elaborated separately and compared with previous studies. Also, more citations have been added which support our discussion of results. Furthermore, values of oxidation parameters have been discussed in comparison of standard values or acceptable range of values which indicate extent of oxidation or quality of oils. Error bars have been inserted in all the figures and the ANOVA was applied and different alphabets are mentioned in the figures showing significant (p ≤ 0.05) different among various storage conditions at day 90.

**Comment 6: Conclusion**

The conclusion should be revised. In lines 339-343, the authors did not determine or compare the oxidative stability values among the oil types, so authors should not conclude whether SBO is the most unstable; instead, authors should focus on answering the main objectives. If authors would like to conclude or provide this information, authors should statistically compare these values among the oil types.

Author’s response: We have revised this section accordingly and conclude only about main objectives instead of comparison among oils.

**Reviewer #2**

**Comment 1:**

Total Phenolic Contents: one time was not enough to fully extract the phenolic compounds by 80% methanol and hexane. Three times might be better. In addition, the concentration of Folin was missing. Detailed information may be important for other researchers to repeat your tests and data.

Author’s response: Thank you so much for noticing this point. The 3 batches of extractions were performed and combined but did not mention in the text. Now this information is added to the text. here so, we have made the correction about this mistake. Moreover, the concentration of Folin reagent used during this study has also been updated in the main text.

**Comment 2:**

The determination conditions by HPLC should be presented clearly. If possible, please provided the HPLC chromatogram

Author’s response: A detailed method and HPLC condition is given now in the text and a typical chromatogram showing separation of tocols has been provided in the main manuscript.
